# Large Neurofibroma of the Face

**Published:** 2015-06-29

**Authors:** Vasanth S. Kotamarti, Adam M. Feintisch, Ramazi O. Datiashvili

**Affiliations:** Division of Plastic Surgery, Department of Surgery, Rutgers/New Jersey Medical School, Newark

**Keywords:** neurofibromatosis, plexiform, face, periorbital, reconstruction

## DESCRIPTION

A 29-year-old man with neurofibromatosis type 1 (NF1) presented with a giant facial neurofibroma causing significant facial deformity and asymmetry. Ipsilateral paresis of the temporal branch of the facial nerve was evident. He had left-sided blindness secondary to visual axis obstruction. He underwent scalp neurofibroma resection as a child.

## QUESTIONS

1. **What is the etiology of this patient's facial neurofibroma?**
2. **What additional clinical findings might be seen in NF1?**
3. **What are considerations that must be taken into account prior to definitive treatment?**
4. **What are the management options?**

## DISCUSSION

Neurofibromatosis comprises a range of disorders sharing a main feature of benign peripheral nerve sheath tumors.^1^ Ninety percent of cases are NF1, or von Recklinghausen disease.^2^ The remainder consist of neurofibromatosis type 2 and schwannomatosis. Neurofibromatosis type 1 demonstrates autosomal dominant inheritance due to an inactivating mutation of neurofibromin.^1^ Histological examination of tumors demonstrates proliferation of both nerve and local stromal elements. The morphological variants of NF1 are cutaneous, nodular plexiform, diffuse plexiform, and subcutaneous.^3^ Craniofacial involvement is typically of the plexiform variant of NF1.^2^ Plexiform neurofibromatosis is characterized by neural tissue hamartomas that grow along nerve sheaths within the subcutaneous fat or deeper tissues.^1^ It carries a 10% risk of transformation into a malignant peripheral nerve sheath tumor.^4^

Two of 7 cardinal clinical criteria, developed by the National Institutes of Health, must be present for a diagnosis of NF1. These include 6 or more café au lait spots more than 5 mm in diameter in prepubertal and more than 15 mm in diameter in postpubertal individuals, 2 or more Lisch nodules (pigmented iris hamartomas), axillary (Crowe sign) or inguinal freckling, optic glioma, 2 or more neurofibromas of any type or 1 plexiform neurofibroma, a distinctive osseous lesion, or an affected first-degree relative. Tumors may involve underlying fascia, muscle, bone, and potentially viscera leading to systemic symptoms. Subsequent deformities may occur primarily via direct tumor involvement or secondarily by traction on neighboring structures due to gravity.^5^ Hypertrophy of adjacent connective tissue and underlying bone can occur.^3^ Craniofacial lesions may lead to motor and sensory deficits due to cranial nerve disruption.^2^

Because of the cosmetic and functional importance of one's face, patients with craniofacial lesions incur a high risk for psychological disturbances.^5^ Complete tumor resection may not be feasible without risk for facial nerve injury or extensive muscle resection. In addition, as neurofibromas lack a capsule and have a substantial and friable blood supply, dissection is typically tedious with a high risk for substantial blood loss.^6^ Conservative or partial excisions portend a concern of recurrence, whereas more radical excisions may require aggressive reconstruction of soft-tissue defects.^7^ In cases of midface involvement, lateral structures should be addressed prior to midline structures.^5^ Treatment of concomitant cheek and zygomatic lesions with preservation of canthal and levator function may provide superior outcomes in cases of periorbital involvement.^8^ Critical regions for midface reconstruction include the nasolabial region and oral commissures.^5^

As there is no cure for the disorder itself, the treatment of choice for neurofibromas associated with NF1 is surgical resection.^3^ Medical interventions including antihistamines, maturation agents, and antiangiogenic drugs have had inconsistent results. Potential new therapies such as angiotensin-converting enzyme inhibitors and gene therapy are being investigated. Surgical techniques include partial and radical excisions with a variety of reconstruction options: adjacent tissue transfers, tissue expansion, free flap reconstruction, and composite tissue allotransplantation. Suspension using autologous materials may enable tumor ingrowth and recurrence; using a Teflon mesh netting may prevent this.^7^ Composite tissue allotransplantation is a promising approach that permits radical excision with superior functional and cosmetic reconstruction. A handful of cases have been reported worldwide; such procedures must be performed at well-equipped facilities using a multidisciplinary approach due to the significant risks associated with composite tissue allotransplantation. Ultimately, management should focus on anticipatory guidance, genetic counseling, and symptomatic treatment. Although a period of stability may occur, hasty surgical intervention should be avoided, as rapid recurrence may occur early in the clinical course.^3^

In addition to significant facial asymmetry, our patient developed ipsilateral blindness and facial nerve involvement. His large, ptotic lesion, severe brow, and upper lid ptosis led to significant disfigurement and psychological sequelae. After 2 staged resections, canthopexy, browpexy, and adjacent tissue transfer, the patient remains extremely satisfied, with improved symmetry and appearance 1 year postoperatively. Periorbital resection and reconstruction are planned as a next step of treatment.

## Figures and Tables

**Figure 1:**
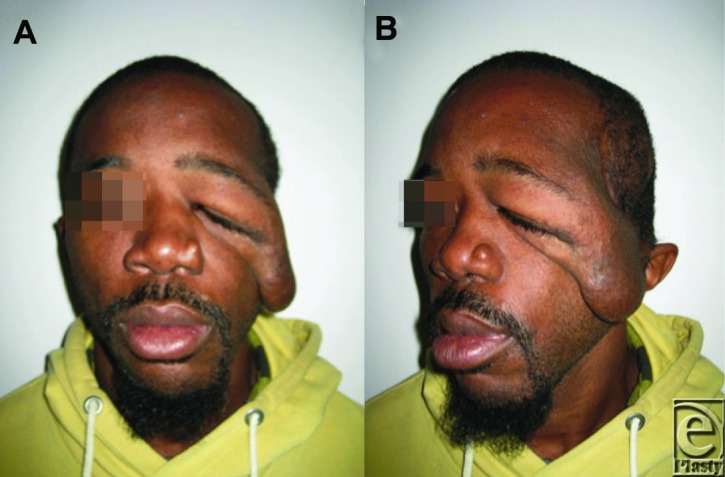
Preoperative (a and b) photographs demonstrating a drastic improvement in facial appearance.

**Figure 2:**
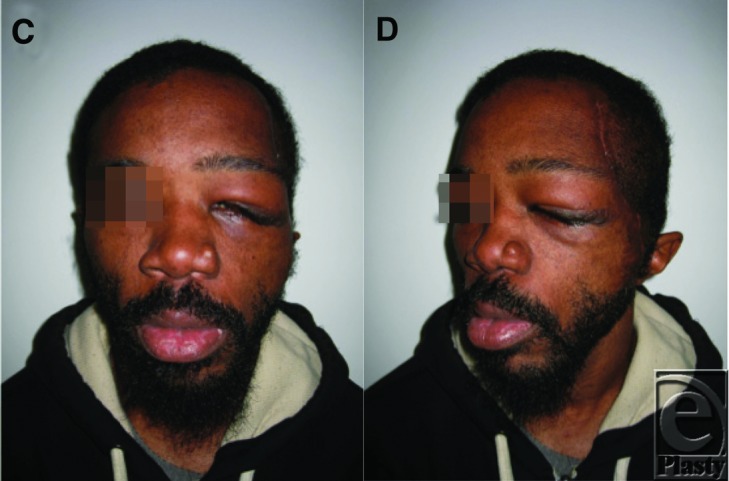
Postoperative (c and d) photographs demonstrating a dramatic improvement in facial appearance.
